# Development and Validation of an MRI-Based Nomogram Model for Predicting Disease-Free Survival in Locally Advanced Rectal Cancer Treated With Neoadjuvant Radiotherapy

**DOI:** 10.3389/fonc.2021.784156

**Published:** 2021-11-15

**Authors:** Silin Chen, Yuan Tang, Ning Li, Jun Jiang, Liming Jiang, Bo Chen, Hui Fang, Shunan Qi, Jing Hao, Ningning Lu, Shulian Wang, Yongwen Song, Yueping Liu, Yexiong Li, Jing Jin

**Affiliations:** ^1^ Department of Radiation Oncology, National Cancer Center/National Clinical Research Center for Cancer/Cancer Hospital, Chinese Academy of Medical Sciences and Peking Union Medical College, Beijing, China; ^2^ Department of Imaging, National Cancer Center/National Clinical Research Center for Cancer/Cancer Hospital, Chinese Academy of Medical Sciences and Peking Union Medical College, Beijing, China; ^3^ Department of Radiation Oncology, National Cancer Center/National Clinical Research Center for Cancer/Cancer Hospital & Shenzhen Hospital, Chinese Academy of Medical Sciences and Peking Union Medical College, Shenzhen, China

**Keywords:** rectal neoplasms, neoadjuvant therapy, magnetic resonance imaging, nomograms, prognosis

## Abstract

**Objectives:**

To develop a prognostic prediction MRI-based nomogram model for locally advanced rectal cancer (LARC) treated with neoadjuvant therapy.

**Methods:**

This was a retrospective analysis of 233 LARC (MRI-T stage 3-4 (mrT) and/or MRI-N stage 1-2 (mrN), M0) patients who had undergone neoadjuvant radiotherapy and total mesorectal excision (TME) surgery with baseline MRI and operative pathology assessments at our institution from March 2015 to March 2018. The patients were sequentially allocated to training and validation cohorts at a ratio of 4:3 based on the image examination date. A nomogram model was developed based on the univariate logistic regression analysis and multivariable Cox regression analysis results of the training cohort for disease-free survival (DFS). To evaluate the clinical usefulness of the nomogram, Harrell’s concordance index (C-index), calibration plot, receiver operating characteristic (ROC) curve analysis, and decision curve analysis (DCA) were conducted in both cohorts.

**Results:**

The median follow-up times were 43.2 months (13.3–61.3 months) and 32.0 months (12.3–39.5 months) in the training and validation cohorts. Multivariate Cox regression analysis identified MRI-detected extramural vascular invasion (mrEMVI), pathological T stage (ypT) and perineural invasion (PNI) as independent predictors. Lymphovascular invasion (LVI) (which almost reached statistical significance in multivariate regression analysis) and three other independent predictors were included in the nomogram model. The nomogram showed the best predictive ability for DFS (C-index: 0.769 (training cohort) and 0.776 (validation cohort)). It had a good 3-year DFS predictive capacity [area under the curve, AUC=0.843 (training cohort) and 0.771 (validation cohort)]. DCA revealed that the use of the nomogram model was associated with benefits for the prediction of 3-year DFS in both cohorts.

**Conclusion:**

We developed and validated a novel nomogram model based on MRI factors and pathological factors for predicting DFS in LARC treated with neoadjuvant therapy. This model has good predictive value for prognosis, which could improve the risk stratification and individual treatment of LARC patients.

## Introduction

The current standard treatment for locally advanced rectal cancer (LARC) is neoadjuvant therapy (NAT) followed by total mesorectal excision (TME) and postoperative adjuvant chemotherapy (ACT) (1). However, because of the heterogeneity that exists in LARC patients, the prognosis of patients in the same treatment model may be considerably different, which shows that TNM staging is not able to accurately predict clinical prognosis for rectal cancer (2).

Considering the importance of risk stratification and prognosis prediction, a stable and computationally simple prognostic model is necessary for clinical applications. Although several models have been established, they are mostly based on pathological factors (3, 4). Magnetic resonance imaging (MRI) is an effective imaging modality whose assessment has important clinical value and should be considered for inclusion in prognostic models (5–7). Due to its soft-tissue contrasts and high spatial resolution, standardized and comprehensive pretreatment MRI assessment is of great significance. According to the European Society for Medical Oncology (ESMO) guideline, structured MRI reports should include the tumor location, primary tumor stage (MRI-T stage, mrT), node stage (MRI-N stage, mrN), extramural vascular invasion (EMVI) and mesorectal fascia (MRF), which demonstrates that pretreatment MRI factors are prognostic factors for LARC (1). Model construction based on factors of pre-neoadjuvant MRI factors and post-treatment pathological findings is expected to provide a more comprehensive evaluation to prognosis. In this study, we will build a model based on standardized structural pre-treatment MRI evaluation and pathological results.

In the present study, we aimed to develop and validate a model predictive of disease-free survival (DFS) after neoadjuvant radiotherapy for LARC. We combined pretreatment MRI and pathological factors to stratify the prognosis of LARC patients treated with neoadjuvant radiotherapy, and we believe the MRI-based nomogram model will help clinicians evaluate the risk stratification of patients and guide follow‐up plans.

## Materials and Methods

### Patients and Clinical Characteristics

We retrospectively analysed patients with LARC (mrT3–4 and/or mrN+) who had undergone neoadjuvant radiotherapy from March 2015 to March 2018 at our institution. All of these patients had received neoadjuvant radiotherapy before rectal cancer surgery. The clinical data were retrospectively collected. The inclusion criteria were as follows: (1) patients were pathologically diagnosed with primary adenocarcinoma; (2) patients underwent pretreatment high-definition MRI evaluation and were staged as LARC; (3) patients received neoadjuvant radiotherapy; and (4) patients did not have any other malignancy. The exclusion criteria were as follows: (1) patients with synchronous distant metastasis; (2) patients with insufficient MRI quality; (3) patients who did not complete neoadjuvant radiotherapy; and (4) patients with a lack of operative pathology information. Ultimately, a total of 233 patients who met these criteria were included for analysis. The patients were sequentially divided into two cohorts (training cohort and validation cohort) at the time of pretreatment MRI. The grouping ratio was 4:3, with 133 patients in the training cohort and 100 patients in the validation cohort. The baseline clinical characteristics were also collected.

### MRI and Image Evaluation

Pretreatment MRI was performed within 4 weeks before the start of neoadjuvant therapy. MRI was performed with a 3.0 T MRI scanner (Signa HDx, General Electrics, Milwaukee, WI, USA). The MR imaging protocols included axial, sagittal, and coronal T2-weighted (T2W) images, axial T2-weighted sequences with fat saturation, and axial T1-weighted and diffusion-weighted imaging (DWI) images. The MR imaging parameter details are presented in the Supplementary material (**Supplement Table S1**). The structured report of MRI factors (**Supplement Table S2**) was evaluated by two senior radiologists, and the results were then compared to reach a final consensus. Both radiologists were blinded to all clinical and histopathological information. The MRI factors of rectal cancer included the tumor location (classified according to the distance from the anal verge to the distal tumor edge on sagittal T2W imaging), mrT (assessment of T staging according to the 7th edition American Joint Committee of Cancer (AJCC) staging system) (8), mrN (assessment of nodal staging according to the European Society of Gastrointestinal and Abdominal Radiology (ESGAR) consensus) (9), MRF status (distance of mesorectal fascia from tumor less than or equal to 1 mm) (10), and EMVI (the definition of MRI-EMVI (mrEMVI) refers to Smith’s scoring system) (11).

### Treatment and Pathologic Assessment

In the present retrospective study, NAT consisted of two modalities: concurrent chemoradiation therapy (CRT) and short-term radiotherapy (5 Gy x 5). For CRT, patients received 50 Gy/25 F radiation concurrently with capecitabine (825 mg/m2 twice daily during radiotherapy). For short-term radiotherapy, patients received short-term radiotherapy (5 Gy x 5) followed by 4 courses of CAPOX (capecitabine (1000 mg/m2 twice daily) combined with oxaliplatin (130 mg/m2 every 3 weeks) at 7–14 days after the completion of radiation. TME surgery was performed after a median time interval of 6–8 weeks after the completion of NAT. Pathologic staging according to the 7th edition AJCC staging system was determined by examination of the surgical specimen (8). Pathological assessment included the evaluation of TNM stage, lymphovascular invasion (LVI), perineural invasion (PNI) and tumor regression grade (TRG). The TRG was reported according to Dworak grading (12). According to the TRG, the patients were divided into a poor responder group (TRG 3–5) and a good responder group (TRG 1–2).

### Statistical Analysis

Statistical analyses were performed using R statistical software (R version 3.6.3). Univariate survival analysis was performed using the Kaplan–Meier method. Multivariate analyses were analyzed by the Cox proportional hazards regression model (survival package), for predictor selection, the stepwise elimination method was used. The correlations between the selected factors were assessed by Pearson’s or Spearman’s coefficient. The nomogram construction was performed by the rms package. The nomogram model was evaluated by Harrell’s concordance index (C-index), receiver operating characteristic (ROC) curve analysis (timeROC package) and calibration curves. The MRI-based nomogram model was compared with the results of two previously published nomogram prediction models based on pathological factors [Model A comes from Li et al., model B comes from Wei et al. (4, 13)]. The optimal cut-off value of the nomogram group was determined according to the highest χ2 value defined by the log-rank test and Kaplan-Meier survival analysis using X-tile (Rimm Laboratory, Yale University, version 3.6.1) (14). According to the method of Vickers et al. (15, 16), the clinical utility of the model was evaluated with decision curve analysis (DCA). DCA explores the clinical benefit of the nomogram model by calculating the net benefit of each decision strategy at each threshold probability (17). The primary outcome was DFS, which was measured from the time of the initial imaging diagnosis until the occurrence of a DFS event (including death, local recurrence or metastasis) or censoring. A two-sided P value <0.05 was considered statistically significant.

## Results

### Clinical Characteristics

The baseline characteristics of the training and validation cohorts are summarized in **Table 1**. The median age was 58 years (range: 20–80 years) in the training cohort and 57 years (range: 31–74 years) in the validation cohort. A total of 73 (54.9%) training cohort patients and 64 (64.0%) validation cohort patients had lesions within 5 cm of the anal verge. Most of the patients in both cohorts had mrT3 and mrN+ disease. The positive rates of MRF involvement and EMVI were 60.2% (80/133) and 54.9% (73/133) in the training cohort and 65.0% (65/100) and 54.0% (54/100) in the validation cohort, respectively. No significant differences were found in the pretreatment MRI and pathology factors were observed between the training and validation cohorts except for TRG (Dworak).

**Table 1:** Characteristics of the patients in the training and validation cohorts.

Characteristics	Training cohort (n =133)	Validation cohort (n =100)	P
Gender			
Male	88 (66.2)	71 (71.0)	0.482
Female	45 (33.8)	29 (29.0)	
Age at diagnosis (y), median (range)	58 (20-80)	57 (31-74)	0.320
Distance to the anal verge (cm)			
5.1-10	60 (45.1)	36 (36.0)	0.162
<5	73 (54.9)	64 (64.0)	
MRI T stage			
cT2	4 (3.0)	0 (0)	0.091
cT3	110 (82.7)	91 (91.0)	
cT4	19 (14.3)	9 (9.o)	
MRI N stage			
cN0	23 (17.3)	15 (15.0)	0.837
cN1	65 (48.9)	48 (48.0)	
cN2	45 (33.8)	37 (37.0)	
MRI-Extramural vascular invasion			
Negative	60 (45.1)	46 (46.0)	0.893
Positive	73 (54.9)	54 (54.0)	
Mesorectal fascia involvement			
Negative	53 (39.8)	35 (35.0)	0.450
Positive	80 (60.2)	65 (65.0)	
Clinical stage			
II	23 (17.3)	15 (15.0)	0.639
III	110 (82.7)	85 (85.0)	
Treatment			
Short-term radiotherapy+chemotherapy	39 (29.3)	31 (31.0)	0.782
CRT	94 (70.7)	69 (69.0)	
Pathological T stage			
ypT0	14 (10.5)	14 (14.0)	0.171
ypT1	6 (4.5)	2 (2.0)	
ypT2	40 (30.1)	19 (19.0)	
ypT3	68 (51.1)	63 (63.0)	
ypT4	5 (3.8)	2 (2.0)	
Pathological N stage			
ypN0	89 (66.9)	71 (71.0)	0.743
ypN1	33 (24.8)	23 (23.0)	
ypN2	11 (8.3)	6 (6.0)	
Pathological stage			
0-I	53 (39.8)	34 (34.0)	0.361
II-III	80 (60.2)	66 (66.0)	
Lymphovascular invasion			
Negative	122 (91.7)	86 (86.0)	0.162
Positive	11 (8.3)	14 (14.0)	
Perineural invasion			
Negative	112 (84.2)	75 (75.0)	0.080
Positive	21 (15.8%)	25 (25.0)	
Completeness of resection			
R0	108 (81.2)	83 (82.0)	0.724
R1	25 (18.8)	17 (17.0)	
TRG (Dworak)			
TRG 1	10 (7.5)	18 (18.0)	0.038
TRG 2	46 (34.6)	38 (38.0)	
TRG 3	54 (40.6)	27 (27.0)	
TRG 4	23 (17.3)	17 (17.0)	

Short-term radiotherapy,5 Gy x 5; CRT, chemoradiotherapy; MRI, magnetic resonance imaging; TRG, tumor regression grade.

### Univariate and Multivariate Cox Regression of the Training Cohort

Univariate analyses were performed to identify clinical variables that were significantly associated with DFS in the training cohort. As shown in **Table 2**, MRI N stage and mrEMVI, pathological T stage(ypT), pathological N stage (ypN), pathological stage, LVI, PNI, completeness of resection and TRG were associated with DFS (P value < 0.05). Variables that were significant (P value < 0.05) in the univariable analysis in the training cohort were included in the multivariable analysis. Finally, only three factors (mrEMVI, ypT stage and PNI) remained independent prognostic factors for DFS (**Table 2**).

**Table 2:** Univariate and multivariate analyses of DFS by pretreatment MRI and pathological factors based on the training cohort.

Variable	Univariate analysis	*P*	Multivariate analysis	*P*
	3-year DFS (95% CI)		Hazard ratio (95% CI)	
**Tumor location**				
Mid	64.3 (53.0-77.9)	0.737	NA	
Low	69.6 (59.7-81.1)			
**MRI T stage**				
cT2	75.0 (97.3-100.0)	0.644	NA	
cT3	69.4 (61.1-78.7)			
cT4	52.6 (34.3-80.6)			
**MRI N stage**				
cN0-1	75.9 (67.4-85.4)	0.013	0.992 (0.486-2.023)	0.982
cN2	50.2 (37.4-67.5)			
**MRI-Extramural vascular invasion**				
Negative	79.7 (70.1-90.7)	0.003	2.422 (1.238-4.741)	0.010
Positive	58.9 (45.8-69.4)			
**Mesorectal fascia involvement**				
Negative	71.3 (60.0-84.7)	0.205	NA	
Positive	64.7 (52.9-74.7)			
**Clinical stage**				
II	81.4 (66.4-99.7)	0.052	NA	
III	64.1 (55.7-73.8)			
**Treatment**				
Short-term radiotherapy+chemotherapy	66.7 (53.4-83.2)	0.939	NA	
CRT	67.6 (58.7-77.9)			
**Pathological T stage**				
ypT0-2	90.6 (83.0-98.8)	<0.001	3.805 (1.371-10.559)	0.010
ypT3-4	51.6 (41.6-64.1)			
**Pathological N stage**				
ypN0	79.4 (71.4-88.4)	<0.001	1.727 (0.838-3.182)	0.138
ypN1-2	42.8 (30.2-60.7)			
**Pathological stage**				
0-I	90.6 (83.0-98.8)	<0.001	NA	
II-III	51.6 (41.6-64.1)			
**Lymphovascular invasion**				
Negative	71.5 (63.9-80.2)	<0.001	2.248 (0.966-5.231)	0.060
Positive	18.2 (0.0-40.9)			
**Perineural invasion**				
Negative	74.4 (66.6-83.1)	<0.001	2.923 (1.496-5.231)	0.002
Positive	28.6 (14.5-56.2)			
**Completeness of resection**				
R0	69.9 (61.7-79.2)	0.023	1.079 (0.539-2.161)	0.830
R1	55.4 (38.8-79.1)			
**TRG**				
TRG 3-4	75.0 (65.9-85.4)	0.019	0.884 (0.469-1.666)	0.702
TRG 1-2	56.3 (44.5-71.2)			

TRG, tumor regression grade; NA, Not Available.

### Prognostic Nomogram for DFS

**Figure 1:**
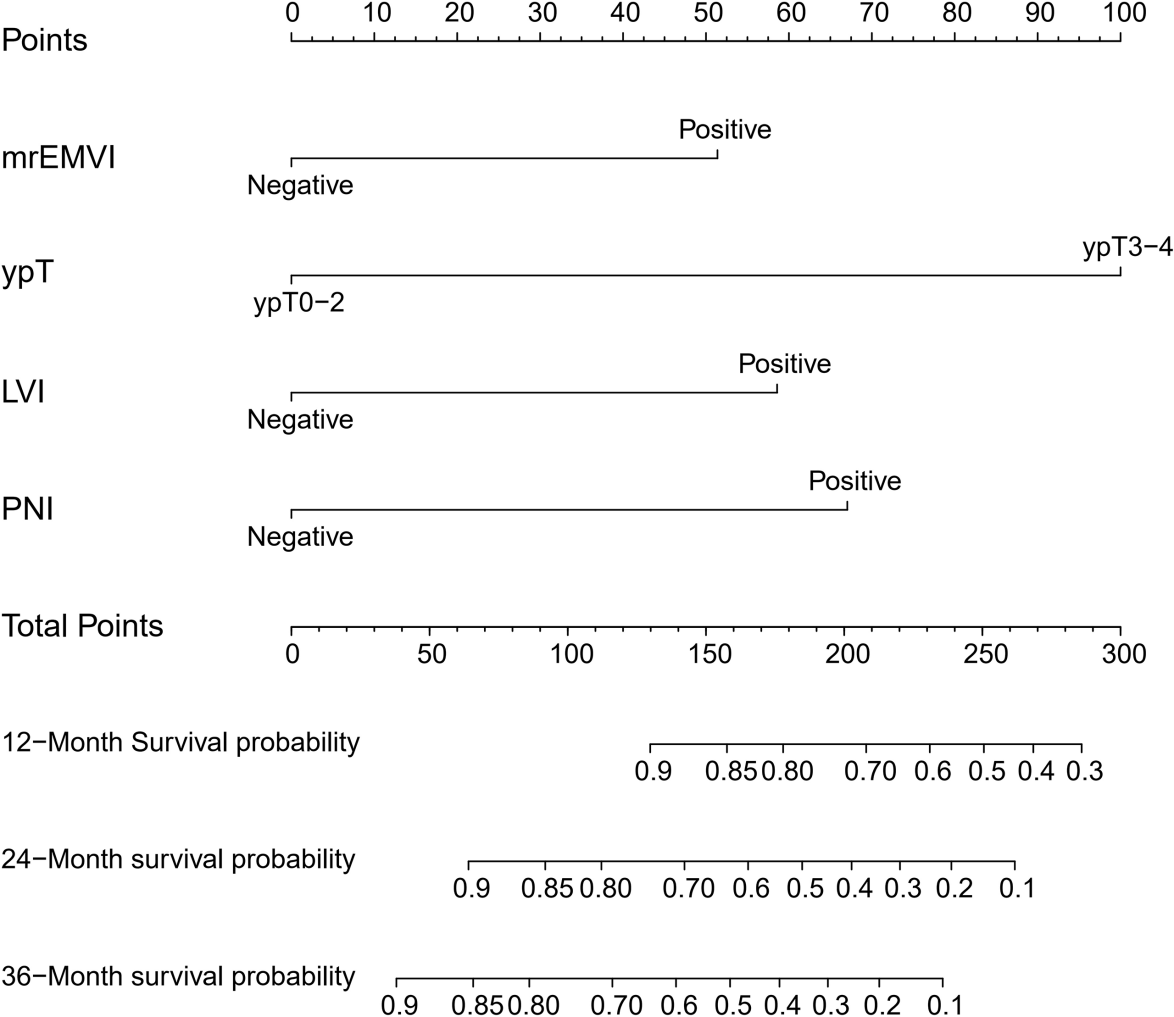
Prognostic nomogram for DFS: The nomogram to predict DFS was developed in the training cohort, and MRI-detected extramural vascular invasion (mrEMVI), ypT, perineural invasion (PNI) and lymphovascular invasion (LVI) were incorporated in the nomogram.

Considering the number of events (n=46) in the training cohort, LVI (which was found to be significant in univariate regression analysis and close to reaching statistical significance in multivariate regression analysis) and three other independent predictors were included in the nomogram model. The Spearman’s correlation coefficients between the selected factors were all less than 0.3. The nomogram for predicting the DFS probabilities of patients at 1, 2, and 3 years is shown in **Figure 1**. In the nomogram, ypT stage was the largest contributor to DFS prognosis, followed by the PNI, LVI and mrEMVI status. Each prognostic factor was given a score on the point scale. By adding the scores of all the selected prognostic factors and locating them on the total point scale, a straight line could be drawn to determine the 1-, 2-, and 3-year DFS probabilities.

### Validation of the Nomogram

**Figure 2:**
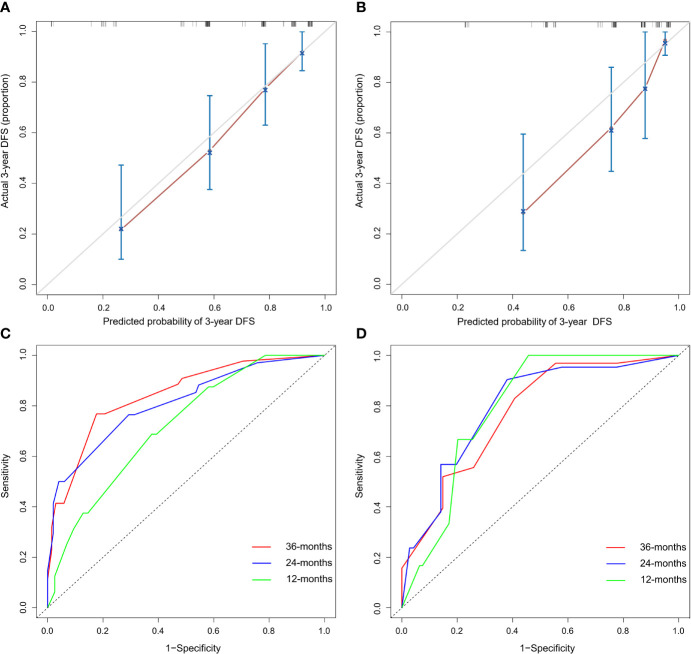
Evaluation of the prognostic nomogram. **(A–D)** Calibration curves for the nomogram in the training cohort **(A)** and validation cohort **(B)**. The x-axis shows the predicted probability of a DFS event. The y-axis shows the actual DFS outcome. **(C, D)** Receiver operating characteristic (ROC) curve for the nomogram in the training cohort **(C)** and validation cohort **(D)**; the AUCs for 1-, 2- and 3-year DFS prediction were 0.720 (95% CI: 0.601–0.839), 0.810 (95% CI: 0.723–0.897) and 0.843 (95% CI: 0.770–0.916) in the training cohort and 0.793 (95% CI: 0.681–0.906), 0.795 (95% CI: 0.693–0.897) and 0. 771 (95% CI: 0.648–0.893) in the validation cohort, respectively.

The C-indices of the nomogram for DFS prediction were 0.769 (95% confidence interval (CI): 0.702–0.837) and 0.776 (95% CI: 0.700–0.853) in the training and validation cohorts, respectively. The calibration plots showed that the probabilities predicted by the nomogram were consistent with the actual probabilities of DFS at 3 years in the training cohort and validation cohort (**Figures 2A, B**). The nomogram yielded an area under the curve (AUC) of 0.843 (95% CI: 0.770–0.916) in the training cohort and 0.771 (95% CI: 0.648–0.893) in the validation cohort (**Figures 2C, D**), which showed that it was more sensitive than the traditional staging system and pathological factor model (**Table 3**).

**Table 3:** Comparison of the nomogram model and other staging systems in terms of the C-index and AUC.

Variables	Training cohort	Validation cohort	Training cohort	Validation cohort
	C-index (95%CI)	C-index (95%CI)	AUC for 3-year DFS (95%CI)	AUC for 3-year DFS (95%CI)
**MRI T stage**	0.553 (0.492-0.615)	0.530 (0.466-0.593)	0.553 (0.476-0.629)	0.535 (0.452-0.619)
**MRI N stage**	0.608 (0.533-0.683)	0.504 (0.414-0.595)	0.633 (0.535-0.730)	0.591 (0.459-0.723)
**Clinical TNM stage**	0.519 (0.480-0.559)	0.511 (0.414-0.641)	0.543 (0.483-0.604)	0.507 (0.413-0.602)
**Pathological T stage**	0.666 (0.606-0.727)	0.676 (0.616-0.735)	0.723 (0.646-0.798)	0.707 (0.608-0.806)
**Pathological N stage**	0.635 (0.564-0.707)	0.677 (0.587-0.767)	0.682 (0.593-0.771)	0.720 (0.604-0.836)
**Pathological TNM stage**	0.529 (0.445-0.613)	0.527 (0.414-0.641)	0.558 (0.453-0.664)	0.592 (0.445-0.739)
**Model A**	0.655 (0.578-0.732)	0.674 (0.577-0.772)	0.714 (0.606-0.822)	0.742 (0.615-0.865)
**Model B**	0.684 (0.609-0.758)	0.625 (0.529-0.720)	0.738 (0.631-0.849)	0.654 (0.483-0.825)
**Nomogram model**	0.769 (0.702-0.837)	0.776 (0.700-0.853)	0.843 (0.770-0.916)	0.771 (0.648-0.893)

AUC, area under the curve. Model A: based on pathological T stage and N stage. Model B: based on pathological TNM stage and perineural invasion.

### Performance of the Nomogram in Stratifying the Risk of Patients

**Figure 3:**
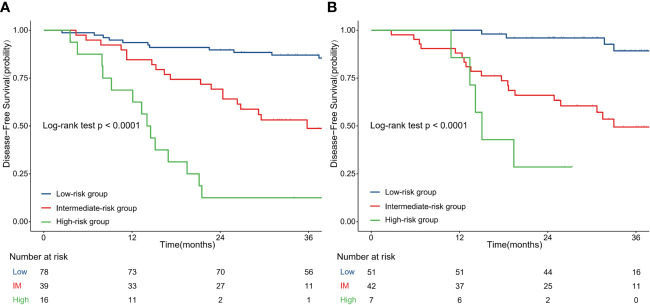
Disease-free survival curves according to patient risk stratification: Survival curves stratified by the nomogram model in the training cohort **(A)** and validation cohort **(B)**; IM, intermediate.

The optimal cut-off value of the nomogram score group was defined by X-tile. Based on the cut-off value of the nomogram score in the training cohort, we divided the patients in the training and validation cohorts into three groups, and the prognosis of each group was significantly different, as shown in **Figure 3**. The median follow-up times were 43.2 months (13.3–61.3 months) and 32.0 months (12.3–39.5 months) in the training and validation cohorts. In the training cohort, the 3-year DFS rates (95% CI) were 87.0% (79.9–94.9) for the low-risk group, 48.7% (34.6–68.7) for the intermediate-risk group and 12.5% (3.4–45.7) for the high-risk group (**Figure 3A**), and those in the validation cohort were 89.3% (79.5–100) for the low-risk group, 49.5% (35.2–69.5) for the intermediate-risk group and 19.4% (8.9–92.2) for the high-risk group (**Figure 3B**) (P value < 0.001).

### Evaluation of the Clinical Efficacy of the Nomogram

**Figure 4:**
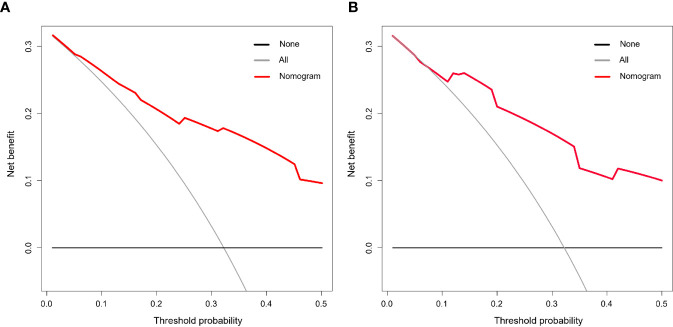
Decision curve analysis (DCA) of the nomogram model. The threshold probability was calculated for the 3-year DFS. **(A)** DCA for the nomogram in the training cohort. **(B)** DCA for the nomogram in the validation cohort. The y-axis represents the net benefit. The x-axis represents the threshold probability. The grey and black lines represent the assumption that all and none of the patients had long-term disease-free survival.

To test the clinical efficacy of the nomogram, DCA was used to assess the clinical utility and net benefit of the nomogram model in the training and validation groups. The net benefit was calculated by adding the true positives and subtracting the false positives. DCA indicated that the use of the nomogram model was associated with a net benefit for the prediction of 3-year DFS compared with the treat-all scheme or the treat-none scheme in the threshold probability range (training group>0.06; validation group>0.08) (**Figure 4**). Here, the all scheme represented the assumption that all patients had long-term disease-free survival, while the none scheme represented the assumption that no patients had long-term disease-free survival.

## Discussion

This single-institution retrospective study evaluated MRI factors and pathological factors as predictors of DFS after neoadjuvant radiotherapy for LARC. mrEMVI, ypT stage, LVI and PNI were predictors of DFS and were used to develop a nomogram. The nomogram showed excellent discrimination and calibration for the individualized prediction of DFS in patients with LARC treated with neoadjuvant radiotherapy and had an AUC of 0.843 (0.770–0.916) for the prediction of DFS at 3 years in the NACT model. We believe that the MRI-based nomogram model will help clinicians evaluate the risk stratification of the patient treated with neoadjuvant radiotherapy and guide follow‐up plans.

DFS is widely used as the endpoint in numerous randomized controlled trials of neoadjuvant treatment for rectal cancer (18–20). DFS is easier to obtain as an endpoint than overall survival (OS) but also requires long-term follow-up. To simplify the prediction of prognosis, pCR and yp0-I stage have been evaluated as long-term outcome surrogate endpoints in previous studies, but the predictive validity was not satisfactory (21). The evaluation of prognosis solely based on pretreatment or posttreatment factors may not be comprehensive and accurate. The neoadjuvant rectal score (NAR) score (formula: 5 ypN–3[cT–ypT] +12]²/9.61) is widely used as a prognostic surrogate endpoint and includes both pretherapeutic and posttherapeutic factors (22, 23). However, the NAR score has limitations in that only considers the downstaging of T stage and ypN stage, which can be further improved. To compensate for this limitation, we performed model construction and evaluation based on high-definition MRI and detailed pathology assessment.

Based on the results of the prognostic analysis and number of DFS events (n=46) in the training cohort, we selected one MRI factor and three pathological factors to be included in the prediction model. Because of its high spatial resolution and soft tissue resolution, MRI has become an important part of the local assessment methods for rectal cancer (24). In accordance with the ESMO guidelines, the MRI factors were evaluated in the present study. mrEMVI status was an independent prognostic factor included in our prognostic model. mrEMVI is an important prognostic factor for rectal cancer patients treated with NAT (25). During the baseline MRI diagnostic assessment, mrEMVI status can be acquired with high accuracy (AUC=0.788) (6). The presence of mrEMVI positivity correlates significantly with increased risks of distant metastasis and local recurrence in rectal cancer patients (11, 26). The metastasis risk for mrEMVI-positive patients was four-fold higher than that for mrEMVI-negative patients (27), and this clinical factor agrees with the main reason for poor DFS in rectal cancer, which is the occurrence of distant metastases (28). Compared with the other MRI factors, mrEMVI is more sensitive in prognostic prediction for rectal cancer patients treated with NAT. Zhang et al. found that mrEMVI was the only independent predictor for OS, metastasis-free survival and relapse-free survival (P<0.05), which indicated the important value of mrEMVI as a prognostic factor for prognostic models.

In the present study, pathological factors were obtained from the structured pathology report. According to previous studies and the Cox regression analysis results, ypT stage, PNI, and LVI were included in the nomogram model. Pathological T stage is an important part of AJCC cancer staging, which is used for the risk stratification of rectal cancer patients (17). In the era of neoadjuvant treatment, ypT still has important prognostic value (29). In the prospective cohort MERCURY study, survival outcomes showed that the prognostic importance of ypT was independent of the type of treatment received. The 5-year OS, DFS and local recurrence rates were significantly lower in poor ypT than in good ypT response (39% vs. 76%, 38% vs. 84%, and 27% vs. 6%) (29). In colorectal cancer, PNI and LVI are well-known high-risk factors for distant metastasis, and the incidences of PNI and LVI were 24.3% and 14.4%, respectively, in LARC patients treated with concurrent radiochemotherapy (30–32). According to the Swedish colorectal cancer registry database, patients with LVI (hazard ratio (HR)=1.44, p=0.011) and PNI (HR=1.80, p<0.001) had significantly increased risks of recurrence. In multivariate Cox regression analysis, PNI indicated a worse DFS outcome (HR=1.37, p=0.005) (33). Song et al. found that PNI and LVI were poor prognostic factors for LARC patients treated with radiochemotherapy and radical operation. According to the status of LVI and PNI, patients can be divided into four groups: both negative, LVI+ only, PNI+ only, and both positive. There were significant differences in 5-year OS and distant failure-free survival (p<0.001), and the both-positive group had the worst prognosis (34). Therefore, we believe that the inclusion of these pathological factors, which have prognostic value in a neoadjuvant radiotherapy model, could contribute to the accuracy of the predictive model.

A growing number of prognostic nomogram models have been published in recent years, and some models based on pretreatment MRI-factors can be used for the prediction of the response to neoadjuvant therapy and prognosis (35–37). However, only a few studies have specifically focused on the long-term prognosis (DFS) of patients receiving neoadjuvant radiotherapy models (long-term or short-term radiotherapy) combined with TME surgery. Although LARC patients account for the majority of the population in most studies, because the treatment time of patients in the training group is far from the current time, nearly half of the LARC patients did not receive neoadjuvant therapy, the treatment mode includes neoadjuvant radiotherapy combined with surgery or direct surgery. Neoadjuvant radiotherapy for rectal cancer has become the standard treatment strategy for locally advanced rectal cancer (cT3-4, or N+) patients (1), and the confounding of treatment factors will affect the prediction accuracy and applicable population of the model. In this study, patients in both the training cohort and validation cohort were treated with neoadjuvant radiotherapy (NAT) combined with TME surgery, the consist of treatment between the two groups was not statistically different, and the radiation dose, technique, and chemotherapy regimen were also more consistent with current guidelines (1). Therefore, the MRI-based nomogram model from our study is more suitable for the LACR patient population, and the different models complemented each other in predicting the prognosis of patients with rectal cancer in different populations.

Compared with the pretreatment models, our nomogram model includes pretreatment factors and pathological factors, whose evaluation is based on MRI and postoperative pathology reports. Although the addition of pathological factors makes this model unable to evaluate prognosis before NAT, pathological factors have a nonnegligible value in long-term prognosis. Potential heterogeneity (such as the sensitivity of treatment) may lead to limitations despite patients having the same pretreatment factors. Pathological factors can directly reflect the downstaging and sensitivity of NAT. A series of prospective clinical trials have shown that pathological downstaging is closely related to long-term prognosis under neoadjuvant treatment and can be used as a surrogate endpoint (38, 39). Therefore, pathological factors are of great value in prognostic models. With the development of radiomics technologies, MRI-based radiomics models are gradually being used to establish prognostic models (40, 41). Radiomics can extract quantitative features from images, and disease features that cannot be visualized may be identified through radiomic feature analysis (42). Although radiomics shows great potential for application, there are still limitations in terms of the repeatability and reproducibility. A systematic review showed that the repeatability of shape metrics and textural features was lower than that of first-order features (i.e., histogram-based features) (43). Regarding reproducibility, although feature extraction software packages such as MaZda, PyRadiomics and LifeX have been widely used (44–46), the workflow and data processing of each study are quite different, and there are some unreported details of data analysis. Because of the numerous factors (i.e., image quality and sequence) affecting repeatability and reproducibility, it is necessary to standardize and reform the methodology. However, we look forward to the widespread use of radiomics models in clinical applications. Additionally, nomogram models based on pretreatment MRI and pathological factors are reproducible and stable and still have important clinical value. Following the risk stratification of this model, high-risk patients will have a significantly higher risk of local recurrence and distant metastasis. Individualized surveillance may be more appropriate such as more frequent follow-up computed tomography, MRI or tumor markers. Regarding adjuvant chemotherapy, high-risk patients may benefit from higher-intensity chemotherapy regimens.

This study has several limitations. First, this was a single-centre retrospective study, and the size of the sample was relatively small. Second, although we tried to include consecutive patients in the study, a certain degree of selection bias was still unavoidable. Third, because of the sample size, the number of patients in the high-risk group was relatively small, and the prognostic evaluation of the high-risk group may be limited. Therefore, a multicenter prospective study or the high-quality multicenter retrospective data with a larger sample size might be needed to validate and refine the nomogram model.

## Conclusions

In conclusion, we constructed a nomogram that included pretreatment MRI factors and pathological factors and could be conveniently applied for the prediction of DFS in patients with LARC. The nomogram model shows better potential predictive value for prognosis and could improve the risk stratification and individual treatment of LARC patients. Further external validation is warranted to obtain a higher level of evidence for the nomogram before its use in clinical practice.

## Data Availability Statement

The original contributions presented in the study are included in the article/**Supplementary Material**. Further inquiries can be directed to the corresponding author.

## Ethics Statement

The studies involving human participants were reviewed and approved by Cancer Hospital, Chinese Academy of Medical Sciences and Peking Union Medical College. Written informed consent for participation was not required for this study in accordance with the national legislation and the institutional requirements.

## Author Contributions

SC and JiJ contributed conception and design of the study. SC and YT organized the database. SC and NLi performed the statistical analysis. LJ and JuJ were responsible for the evaluation of MRI features. All authors analyzed and interpreted the detailing. SC wrote the first draft of the manuscript. JiJ take final responsibility for this article. All authors contributed to the article and approved the submitted version.

## Funding

The present study was supported by National Natural Science Foundation of China (81871509); Fundamental Research Funds for Central Universities of the Central South University (3332019055); Capital’s Funds for Health Improvement and Research (2020–1–4021).

## Conflict of Interest

The authors declare that the research was conducted in the absence of any commercial or financial relationships that could be construed as a potential conflict of interest.

## Publisher’s Note

All claims expressed in this article are solely those of the authors and do not necessarily represent those of their affiliated organizations, or those of the publisher, the editors and the reviewers. Any product that may be evaluated in this article, or claim that may be made by its manufacturer, is not guaranteed or endorsed by the publisher.
