# Supplementary material for: Development and Validation of an MRI-Based Nomogram Model for Predicting Disease-Free Survival in Locally Advanced Rectal Cancer Treated With Neoadjuvant Radiotherapy
Source: Front Oncol. 2021 Nov 15;11:784156. doi: 10.3389/fonc.2021.784156 (PMC8634258; doi:10.3389/fonc.2021.784156)
Supplement: Supplementary file 1 [file Table_1.docx]

**Table S1. Protocols for the MR imaging sequences**

| Parameter | Oblique T2WI | Sagittal T2WI | Coronal T2WI | T1WI | T2WI/FS | DWI (b=0, 1000) |
| --- | --- | --- | --- | --- | --- | --- |
| TR (ms) | 4800 | 4800 | 4800 | 560 | 5700 | 2,300 |
| TE (ms) | 115 | 115 | 115 | min | 85 | min |
| FOV (mm) | 160 | 240 | 240 | 340 | 340 | 340 |
| Matrix | 256x320 | 256x320 | 256x320 | 288×224 | 288×224 | 128×160 |
| Band-width (kHz) | 41 | 41 | 41 | 41 | 31 | 250 |
| NEX | 4 | 4 | 4 | 2 | 2 | 2 |
| ETL | 21 | 21 | 21 | 4 | 21 | NA |
| Slice thickness (mm) | 3 | 4 | 4 | 5 | 5 | 5 |
| Intersection gap (mm) | 0 | 0.4 | 0.4 | 0.5 | 0.5 | 0.5 |

DWI, diffusion-weighted imaging; ETL, echo train length; FOV, field of view; FS, fat saturation; NA, not available; NEX, number of excitations; TE, echo time; TR, repetition time; T1WI, T1-weighted imaging; T2WI, T2-weighted imaging.

**Table S2. Rectal Cancer Primary Staging MRI Report**

| **Tumor location**：  Location from the anal verge ：rectosigmoid junction □, Rectal □（Hig □,Mid □,low □）  Involved the dentate line：Yes □, No □  Rectal wall ：anterior □, posterior □, left □, right □  **T-category:**  Thickness of the lesions: (cm)  T-stage: T1 □, T2 □, T3 □, T4 □  T3-stage: ≦1mm(T3a) □, 1-5mm(T3b) □, 5-15mm(T3c) □, >15mm (T3d)□  T4-stage: T4a □, T4b □ Organ invasion(T4b):  **Mesorectal fascia involvement (MRF):**  The shortest distance of tumor to MRF: cm  **The status of lymph node:**  Number of suspicious nodes: 0 □,1-3 □,≧4 □  Location of suspicious lymph node: mesorectal □, internal iliac (left □ /right □), presacral □, the root of the inferior mesenteric artery□  **Extramural vascular invasion score:** 1 □, 2 □, 3 □, 4 □  **Other:**  Mucous composition: Yes □, No □  Ascites: Yes □, No □ |
| --- |
| **Clinical stage：** |
